# SALAD: Syringe-based Arduino-operated Low-cost Antibody Dispenser

**DOI:** 10.1016/j.ohx.2023.e00455

**Published:** 2023-07-08

**Authors:** Anh Phuc Hoang Le, Quang Lam Nguyen, Bao Hoai Pham, Thien Hoang Minh Cao, Toi Van Vo, Khon Huynh, Huong Thi Thanh Ha

**Affiliations:** a School of Biomedical Engineering, International University, Ho Chi Minh City, Viet Nam; b Vietnam National University, Ho Chi Minh City, Viet Nam

**Keywords:** Arduino, Lateral Flow Assays, Dispensers, Antibody, Point-of-care, Low-cost

## Abstract

Lateral Flow Assays (LFA) have been one of the most widely adopted technologies in clinical diagnosis over recent years, especially during the COVID-19 pandemic, due to their feasibility, compactness, and rapid readout. However, the precise dispensing of antibodies–a key part of the fabrication process–requires costly line dispenser equipment, which poses a challenge to researchers with limited budgets. This study aims to resolve this key issue by introducing a Syringe-based Arduino-operated Low-cost Antibody Dispenser (SALAD). By utilizing a microneedle, stepper motor-driven syringe pump, and conveyor belt, SALAD can form micro-droplets to create an even band of antibodies. Our evaluation results showed comparable performance between SALAD and a commercialized model – Claremont ALFRD, with SALAD exceeding in affordability and feasibility. SALAD yielded an even signal, uniform bandwidth, and low background noise, yet optimization in the conveyor belt should be considered to enhance stability. With a low manufacturing cost ($200.61) compared to the commercialized models, our model is expected to provide an affordable approach for LFA researchers.

## Graphical abstract

**Figure:**
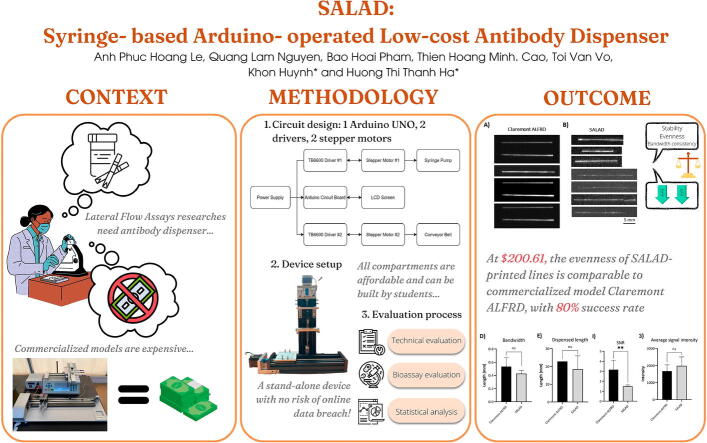

## Specifications table

**Table:** 

Hardware name	Syringe-based Arduino-operated Low-cost Antibody Dispenser – SALAD
Subject area	• Engineering and materials science • Medical • Biological sciences (molecular diagnostic)
Hardware type	• Biological sample handling and preparation
Closest commercial analog	Claremont Bio Automated Lateral Flow Reagent Dispenser - Claremont ALFRD
Open-source license	CC BY 4.0
Cost of hardware	$200.61
Source file repository	http://doi.org/10.17632/sv5y27mzjb.2

## Hardware in context

Point-of-care (POC) diagnostics have become increasingly relevant, especially in the wake of the COVID-19 pandemic, as seen with the proliferation of at-home rapid antigen tests [1]. Most of these tests are based on the lateral flow assay (LFA) principle, one of the POC technologies that have been extensively developed and deployed. The essence of LFA is the capillary force that delivers biofluid samples along the cellulose-based materials and exerts specific interactions with the pre-immobilized reagents at the detection and control lines of the test strips [2]. These docking molecules can be either antibodies, chemically synthesized nucleic acids (aptamers), or molecular beacons with fluorophores and DNA hairpin structures [3]. Compared to traditional testing formats, LFAs are inexpensive, portable, able to produce quick results, and simple to use [3]. In addition, LFAs allow flexible sample inputs, as most biofluids, such as saliva, blood, and urine, are compatible with the format [4]. For these reasons, LFAs have been widely adopted in diagnostic applications such as home testing and infectious disease screening [5]. Other fields, such as environmental testing and food safety, have also employed LFAs to rapidly detect pollutants, chemicals, and toxins [6], [7].

LFAs utilize paper-based materials to fabricate miniature and point-of-care devices for multiple biochemical analyses. The simplified architecture of LFAs test strips includes four parts: a sample pad, a conjugate pad, an absorbent pad, and a detection pad [8]. Regarding the detection pad, the nitrocellulose membrane is considered an optimal candidate because of its hydrophobicity and surface chemistry features, which permit capillary flow [9], [10]. LFAs diagnosis requires the immobilization of detecting antibodies onto the nitrocellulose membrane to form detection and control lines. This procedure is of the essence in the mass production of LFAs test strips, which requires both rapid manufacturing and affordable price, but still ensures industrial-grade quality. For these reasons, automated bioprinting has become prominent in developing LFAs tools - which is also the underlying principle of line dispensers [11], [12].

Current antibodies dispensers work based on the spontaneous interaction between antibodies and the nitrocellulose membrane. Antibodies were hypothesized to bind with nitrocellulose membranes through electrostatic attraction and hydrophobic bonds, allowing them to form connections instantaneously on contact [13], [14], [15]. Nitrocellulose membranes also have a capillary flow that allows liquid to be absorbed instantly and flow in a one-way direction. This capillary flow depends markedly on the porous medium. The capillary forces can also be modified by the fluid characteristics; and by the interaction between the two: the contact angle between the fluid and the porous material [16], [13]. Based on discussed theoretical bases, antibody dispensers were built to dispense reagents directly onto nitrocellulose membranes, leaving them to form bonds passively.

Automated liquid dispensers with state-of-the-art characteristics are essential for components (e.g., chemical, liquid) that require high dispensing precision. In 1998, the principle of commercialized line dispensers was introduced and was later modified in 2009 (which was called the Non-Contact, Pump-Driven Solenoid Dispensers). In the introduced designs, the micro-droplets formation was generated through the produced pressure (gas, hydraulics) coming from the syringe pump, and they are dispensed onto the membrane’s surface to create a line in the form of imbricated drops with the use of a linear motion system [17], [18]. These devices allow precise control over the dispensing quantities, flow rate, droplet size, dispensing patterns such as dot blot or line, and the evenness of liquid throughout the area. Most commercialized Line Dispensers that follow the introduced principle, including the GlucoDot™ Dispense System and DispenseJet DJ-9500 Jet, are solid evidence of the advantages of the original design. However, several devices still have drawbacks, such as difficulty in operating and moving, sophisticated design, and costly (ranging from $3500 to $8500), such as the AD3220™ Aspirate Dispense System. *Hence, with the increasing popularity of utilizing the Lateral Flow Assay in designing and testing a test strip, there is a high demand for an antibody dispenser that is able to promote rapid production of medical test strips and is easily assembled in a university research lab context with a modest budget*. Recently, a model introduced by Han & Shin in 2021 has aimed to meet these requirements by taking advantage of syringe needles with various gauges and using the capillary force created by needle-membrane interaction, which can make a defined line width of antibodies [12]. Although this design proved economical and easy to manufacture, it carries high risks of contamination from an open fluid chamber, and the built materials can be unsuitable for long-term usage.

This study aims to deliver a design that can balance an economic factor with a system that can produce similar results as commercialized models of a line dispenser to encourage the development procedure of Lateral Flow Immunoassays in small laboratory settings. By referring to the binding mechanisms between antibodies and nitrocellulose membrane and the dispensing principle of the introduced commercialized models, this study proposes the design of a dispenser utilizing low-cost instrumentations such as Arduino UNO with stepper motors and TB6600 drivers. A thorough investigation of the efficiency of the Syringe- based Arduino- operated Low-cost Antibody Dispenser (SALAD) was also conducted to evaluate the performance of this design.

## Hardware description

The model introduced in this study (SALAD) combines the working principles of a syringe pump and a conveyor belt. To be more specific, when a syringe is pushed down, the reagent inside the syringe will be dispersed onto the membrane, which is in 90-degree direct contact with the syringe needle. At the same time, the conveyor belt carrying the nitrocellulose membrane will move horizontally, causing the dispensed droplet to spread out into a line on the membrane surface. By having continuous dispensing and the motion occurring synchronously, SALAD can form a continuous uniform line of antibodies. In addition, SALAD takes advantage of low-cost and sustainable materials, such as the linear actuator, stepper motors NEMA 17, and TB6600 Motor Drivers. The Arduino UNO has executive control of all the included components effectively and sustainably. The economical ($200.61) production cost, along with accessible assembly, make SALAD a suitable appliance in university laboratory settings, especially in developing countries.

Research groups can use the design of SALAD to:

• Assemble a similar line dispenser model at a low cost
• Dispense the chemical, antibodies, and antigens onto the membrane surface
• Integrate this model into the research and development procedure of Lateral Flow Immunoassays
• Utilize this model as teaching equipment for engineering, clinical laboratories, or biological laboratories.

**Fig. 1:**
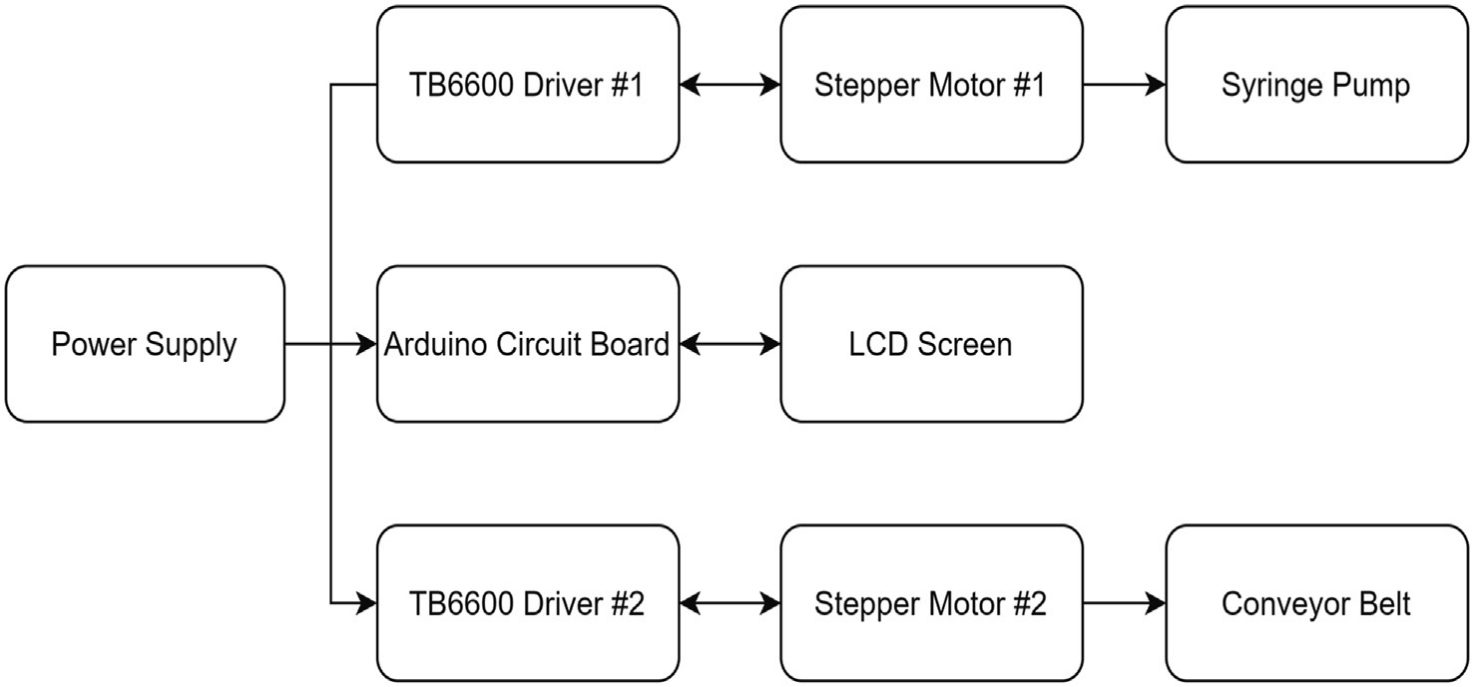
Line Dispenser Design Overview.

In detail, our line dispenser design is described as follows: The system consists of one Arduino UNO circuit board, two stepper motors (going with two-stepper motor actuators), one syringe pump, two TB6600 drivers, four potentiometers, three buttons, and an LCD screen (Fig. 1).

### Stepper motors

Two stepper motors (NEMA 17, length: 42x42mm, step angle: 1.8°, steps per revolution: 200, torque: 0.45Nm) control the × and y-axis movements of the system. The NEMA 17 stepper motor is chosen in this design since it has a high reputation for its reliability and high torque and has been applied in many machine designs: 3D- Printers and CNC machines. The NEMA 17 can also have a smoother operation than the other motors thanks to the assistance of the TB6600 motor drivers. At the top of the dispenser, the first stepper motor pushes down the metal plate and the plunger of the syringe pump, sequentially dispersing the reagent onto the moving membrane. After each run, this stepper motor returns the plate to its original position by reverse rotation. The second motor is connected to an x-axis actuator similar to a conveyor belt. When the second motor rotates, a metal plate carrying the membrane moves horizontally and simultaneously passes the nitrocellulose membrane to the needle tip. The motion of the two motors work synchronized with each other.

### Potentiometer

There are a total of four potentiometers (50 KΩ with three legs) used in our line dispenser model. The first potentiometer controls the total rotating time of the stepper motors; more specifically, the total rotating time starts with the initiation of the y-axis stepper motor. The second potentiometer controls the delaying time between the y and x-axis stepper motors. Two more potentiometers are in charge of the individual speed of each motor.

### LCD Screen

The LCD 16x2 I2C will display four main pieces of information: the rotating time of each motor, rotating speeds, and delay time.

### TB6600- stepper motor drivers

**Fig. 2:**
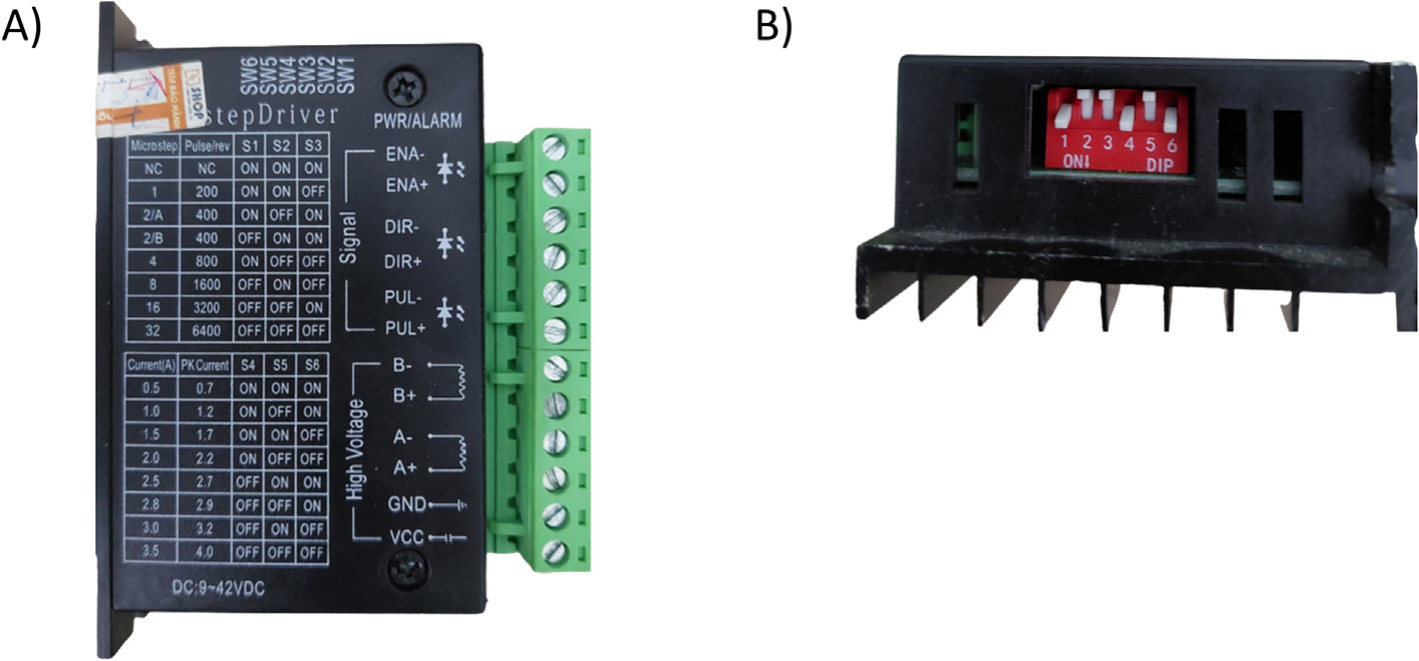
TB6600 Motor Drivers (A). Setup Information and (B). Set Up Switches.

TB6600 Drivers (4.0A – 9 ∼ 42VDC) are used to control the micro-steps and current going through each stepper motor by altering the six switches on the side of the drivers. The TB6600 Motors Drivers can adjust the speed and position of the stepper motors by changing the microstepping resolution, while the drivers (IC TB6600HQ) can control the current using the Pulse-width Modulation voltage. Moreover, the TB6600 motor drivers can ensure the smoothness and stability of the stepper motors while running. The motors were tested at different microsteps and current controls and evaluated for their heat dissipation, rotating speed, and vibration intensity (Fig. 2A). The optimal setting for the SALAD is achieved by setting the micro-step resolution to four, which indicates the microstep/revolution is 800, and the current goes to the stepper motors to 1A. The corresponding driver switches are modified to ON-OFF-OFF for the first three switches and ON-OFF-ON for the remaining three (Fig. 2B).

### Stepper Motors Actuators

Two Stepper Motor Actuators will act as Oy Actuators (for pushing down the Syringe Pump) and Ox Actuators (for controlling the conveyor carrying the membrane). Each Actuator has a lead screw with a diameter of 8 mm and an overall measurement of 320 mm × 78 mm × 48 mm (**length** × **width** × **height**); a C-beam Gantry Plate which is 75 × 75 mm in size; four mini-V wheels with an outside diameter of 15.23 mm and an inside diameter of 9.974 mm; a 4080U Aluminum Linear Guide Slide of 300 mm. All of the mentioned components have been assembled in advance. This length × width × height is suitable in this study for the movement of our designed membrane plate and platter pusher to have enough length to create a thin line on the nitrocellulose membrane.

### Syringe pump

In this experiment, we use the Hamilton® syringe (10 μL/51 mm/26G needle) since we want to dispense 10 μL of antibodies onto the nitrocellulose membrane. If another amount of dispensing liquid volume is required, a different syringe can be replaced to serve the purpose.

## Design files summary

**Table:** 

Design file name	File type	Open-source license	Location of the file
Needle Handle	Fusion 360 (STL)	CC BY 4.0	https://doi.org/10.17632/sv5y27mzjb.2
Stepper Shelf Top	Fusion 360 (STL)	CC BY 4.0	https://doi.org/10.17632/sv5y27mzjb.2
Plater Pusher	Fusion 360 (STL)	CC BY 4.0	https://doi.org/10.17632/sv5y27mzjb.2
Membrane Plate	Fusion 360 (STL)	CC BY 4.0	https://doi.org/10.17632/sv5y27mzjb.2
Left Vertical Frame	Fusion 360 (STL)	CC BY 4.0	https://doi.org/10.17632/sv5y27mzjb.2
Right Vertical Frame	Fusion 360 (STL)	CC BY 4.0	https://doi.org/10.17632/sv5y27mzjb.2
Slide Plating Holder Right	Fusion 360 (STL)	CC BY 4.0	https://doi.org/10.17632/sv5y27mzjb.2
Slide Plating Holder Left	Fusion 360 (STL)	CC BY 4.0	https://doi.org/10.17632/sv5y27mzjb.2
Operation Code	Arduino IDE	CC BY 4.0	https://doi.org/10.17632/sv5y27mzjb.2

**Needle Handle:** Used for positioning the Syringe Pump. The center hole diameter can be changed with respect to the diameter of the Syringe Pump.

**Stepper Shelf Top:** Used for connecting the Stepper Motors with Actuators. There are four holes that can be used to stabilize Stepper Motors with the Stepper Shelf Top and two holes to connect the Stepper Shelf Top with the Actuators.

**Plater Pusher:** Used for pushing down the Syringe Pump. There are two holes that can be used to connect the Plater Pusher with the Oy Actuators.

**Membrane Plate:** Used for holding the Membrane (act as a conveyor belt) in Ox Actuators.

**Left Vertical Frame:** Used for linking the Stepper Shelf Top with the Actuators on the left side.

**Right Vertical Frame:** Used for linking the Stepper Shelf Top with the Actuators on the right side.

**Sliding Plating Holder Right:** Used for holding the Needle Handle on the right side.

**Slide Plating Holder Left:** Used for holding the Needle Handle on the left side.

**Operation Code:** The Arduino Code is used for controlling the SALAH whole operation.

## Bill of materials summary

**Table:** 

Designator	Component	Number	Cost per unit - USD	Total cost -USD	Source of materials	Material type
Syringe Pump	Hamilton® syringe #HAM80075 (10 μL/51 mm/26G needle)	1	47	47	Hamilton	Hardware
Stepper Motor	Stepper Motor NEMA 17 (42x42mm/1.8°/200 steps per rev/0.45Nm)	2	6.41	12.82	Thegioiic	Hardware
Stepper Motor Actuators	T8-320 mm Lead Screw, 300 mm Actuator Structure, Shaft Coupling 5–8 mm, C-beam Gantry Plate with four mini-V wheels)	2	32	64	CuaHangVatTu	Hardware
Arduino UNO	Arduino Uno R3 ATmega328	1	25	25	Hshop	Electronic
Buttons	Two Buttons 6 × 6 × 19 mm and One Button with 12 mm Diameter	3	0.2	0.6	Thegioiic Nshop	Electronic
LCD Screen	LCD 1602 5V with I2C Driver	1	1.95	1.95	Thegioiic	Electronic
Potentiometer	Potentiometer 50 KΩ	4	0.43	1.72	Thegioiic	Electronic
Power Source	Power Adapter 12V-5A	1	4.13	4.13	Thegioiic	Electronic
Stepper Motors Driver	TB6600 Driver (4.0A / 9 ∼ 42VDC)	2	7.63	15.26	Thegioiic	Electronic
CNC Machinery	Aluminum CNC Details (CNC cutting)		20	20	Local	Framework
Iron Corner	Galvanized Iron Corner	Package		5	Shopee	Framework
Iron Screws	Screws M3 and M4	Package		3	Shopee	Framework
Rubber O-ring	Rubber O-ring (CS 3.5 mm, OD 41.5 mm)	Package	0.132	0.132	Local	Framework

The total cost for the Syringe-based Arduino-operated Low-cost Antibody Dispenser is $200.61.

## Build instructions

### Wiring connection

**Fig. 3:**
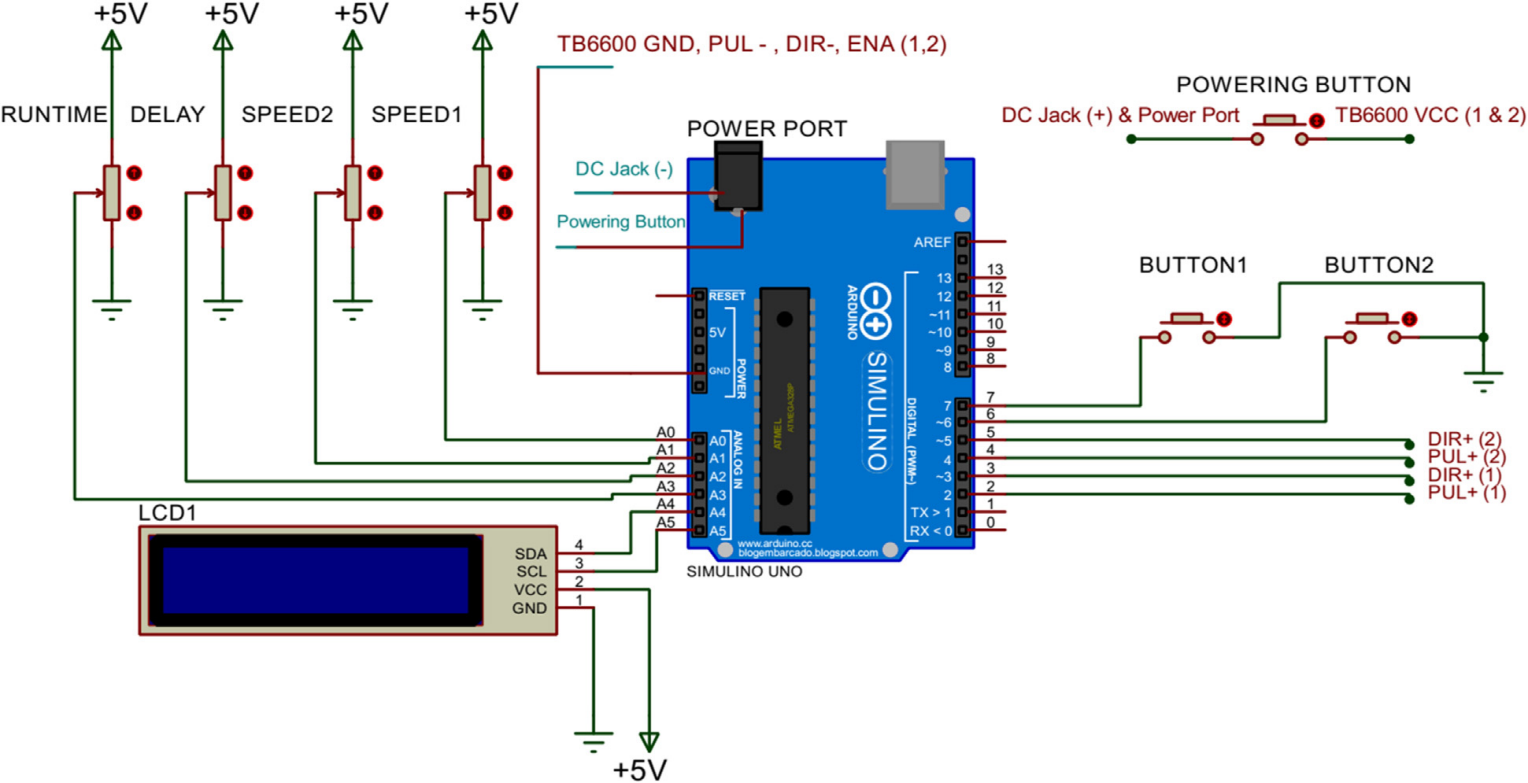
Line Dispenser Circuit Schematic Design.

The potentiometers are connected to pins A0-A4 of the Arduino, with the controlled total rotating time potentiometer to A3, delay time to A2, and speed of the x and y-motor to pins A0 and A1. Each potentiometer is also connected to a 5 V pin and a GND pin. Four pins of LCD I2C are connected to Arduino UNO in the following order: SDA to the A4 pin, SCL to the A5 pin, VCC to the 5 V pin, and GND to the GND pin. Buttons 1 and 2 are connected to pins 7 and 6, respectively. The DIR+ and PUL+, which is the configuration of the TB6600 Motor Driver for direction and pulse, are connected to pins 2 and 3 of Arduino for the TB6600 Driver that controls the first motor and pins 4 and 5 of Arduino of the TB6600 Driver that controls the second motor. On the other hand, A+; A-; B+; B- of TB6600 Drivers are connected to Stepper Motors using Stepper Motors Cables (4 pins JST-PH). Moreover, The GND, PUL- and DIR- and ENA of TB6600 Drivers are connected to each other, and they are wired up to the GND pin of the Arduino. In addition, there is one powering button (supplying the power from the power supply), in which the first leg is connected to the VCC of two TB6600 Motor Drivers and the Power Port of Arduino UNO, while the second leg is connected to the positive side of the DC Jack. Additionally, the negative side of the DC Jack is connected to the Power Port of Arduino UNO. The schematic wiring can be found in Fig. 3.

### Hardware build

#### Connecting two Oy and Ox actuators

**Fig. 4:**
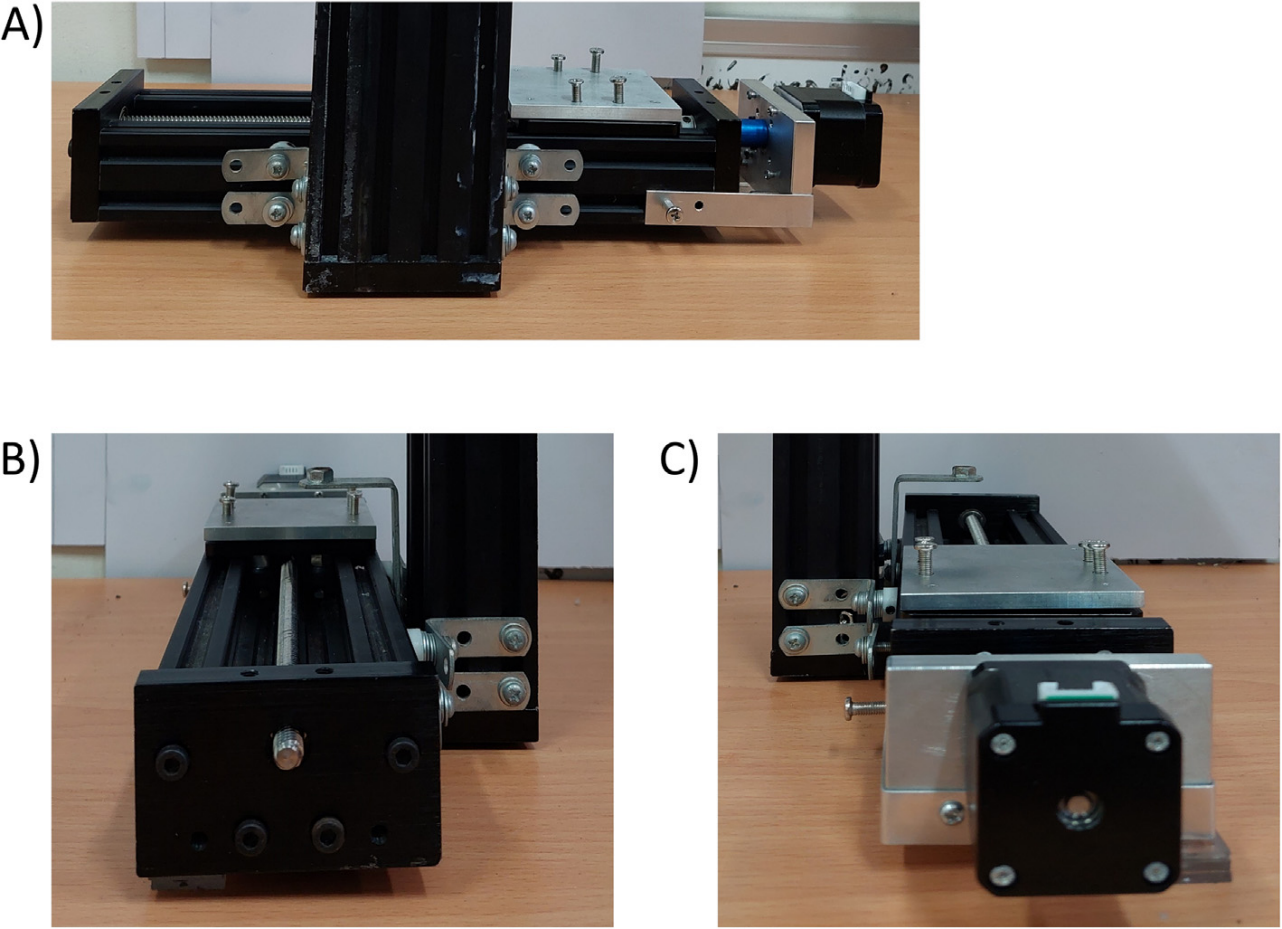
Connecting the Oy and Ox Actuators using Galvanized Irons; (A). Behind Angle; (B) Right-side Angle; (C). Left-side angle.

To connect the two Actuators, two galvanized irons with four M4 screws for each side of the Ox actuators are attached to the Oy actuators (Fig. 4).

#### CNC machining of external materials

**Fig. 5:**
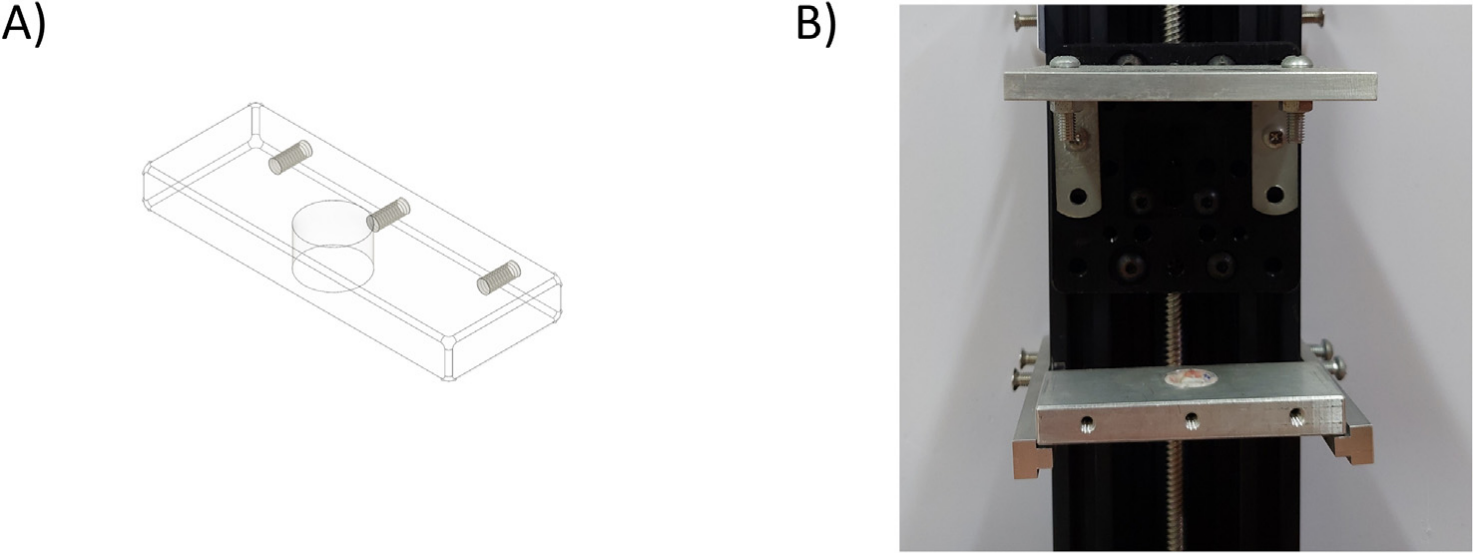
(A) CNC cutting of Needle Handle; (B) Real Images and Position of Needle Handle.

**Fig. 6:**
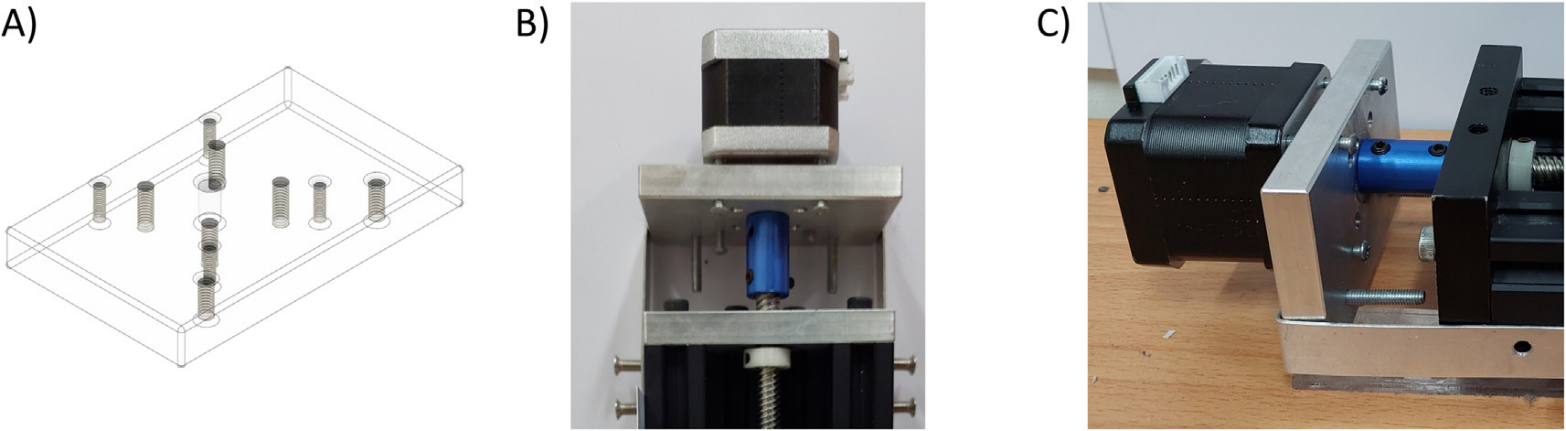
(A) CNC cutting of Stepper Top Shelf; Stepper Shelf Top Real Image and Position in (B) Oy Actuators and (C) Ox Actuators.

**Fig. 7:**
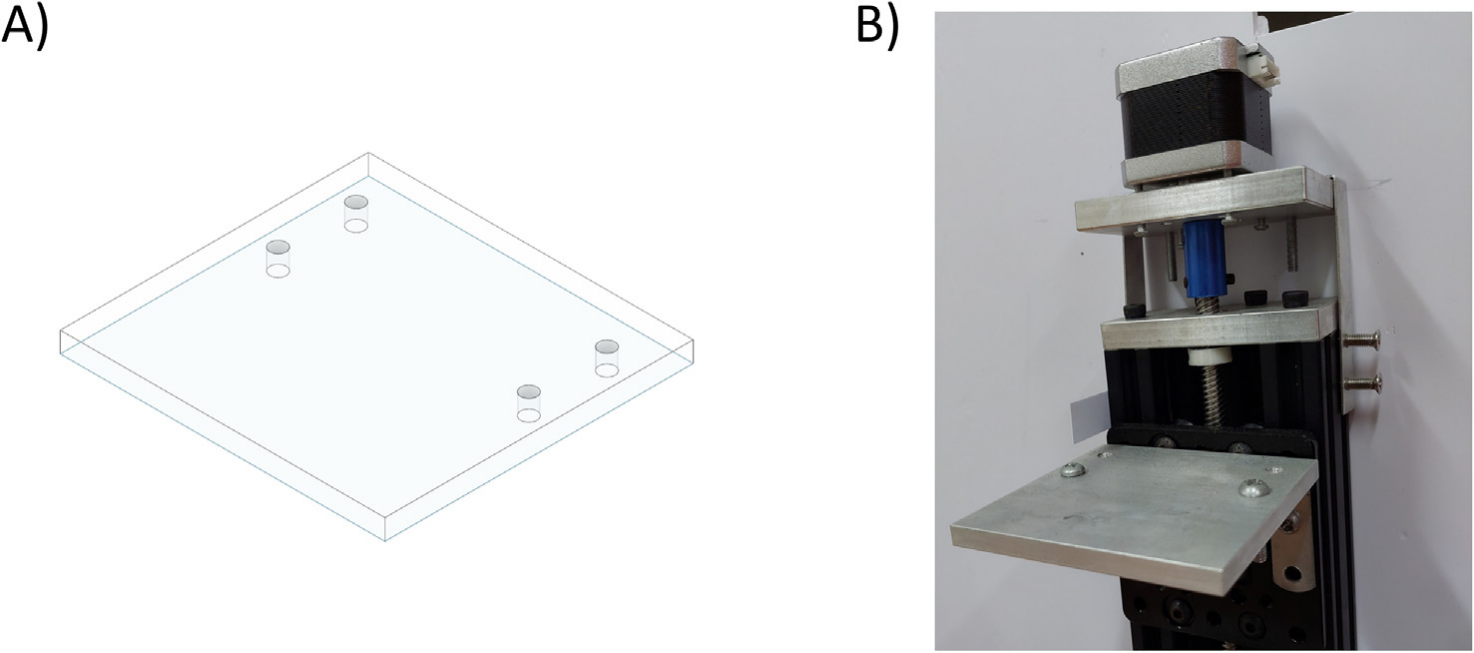
(A) CNC cutting of Plate Pusher; (B) Real Image and Location of Plate Pusher.

**Fig. 8:**
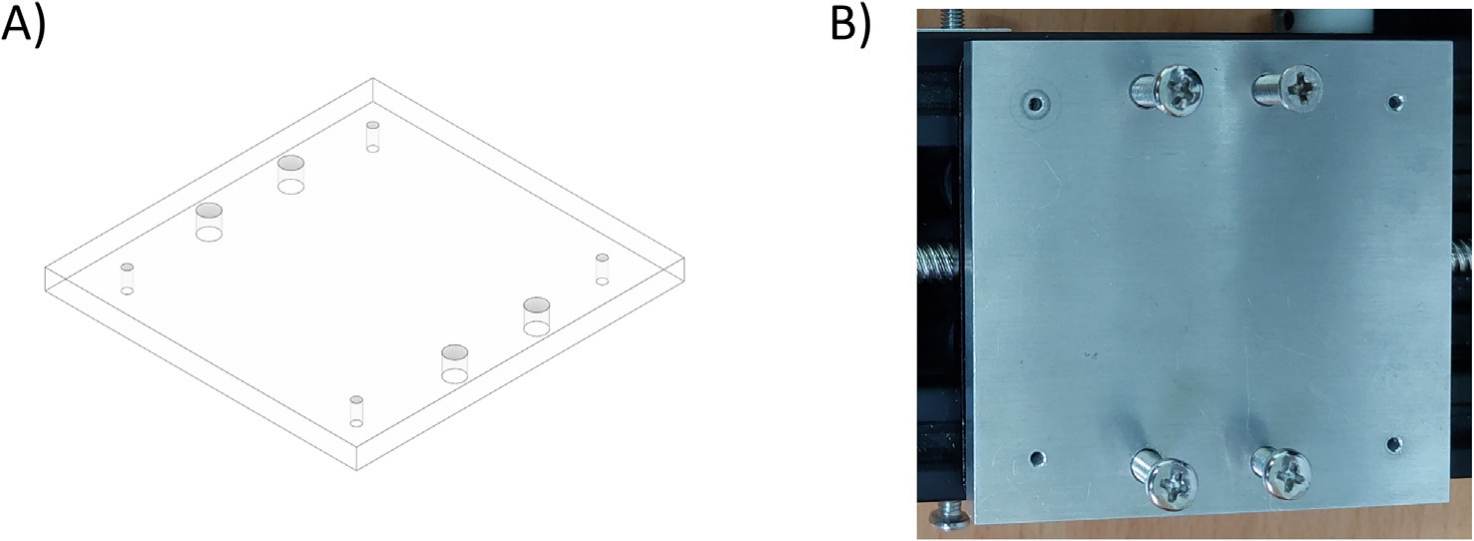
(A) CNC cutting of Membrane Plate; (B) Real Image of Membrane Plate.

**Fig. 9:**
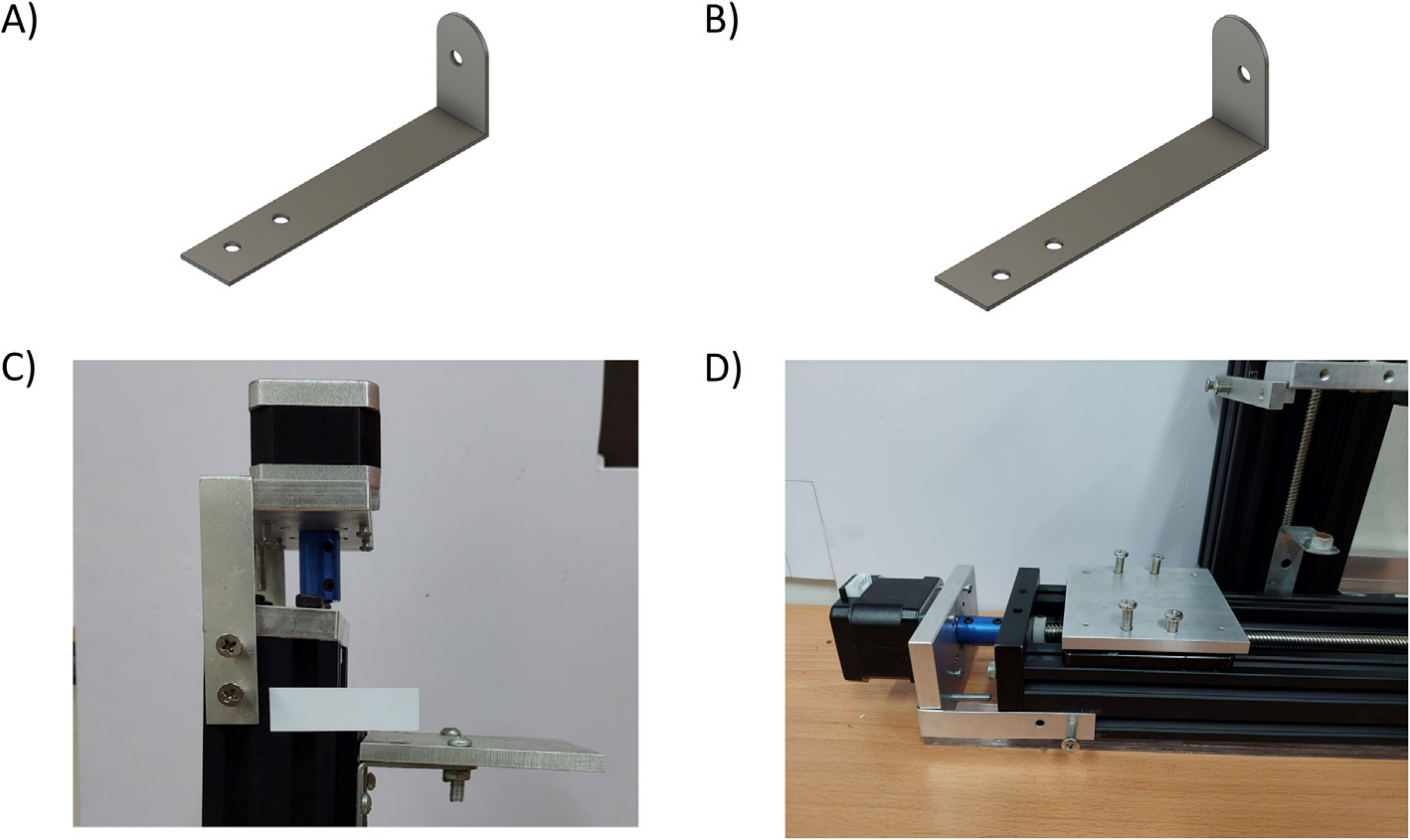
CNC cutting of Vertical Frame on the (A) Left side and (B) Right side; Real Image and Location of Vertical Frame in (C) Oy Actuators and (D) Ox Actuators.

**Fig. 10:**
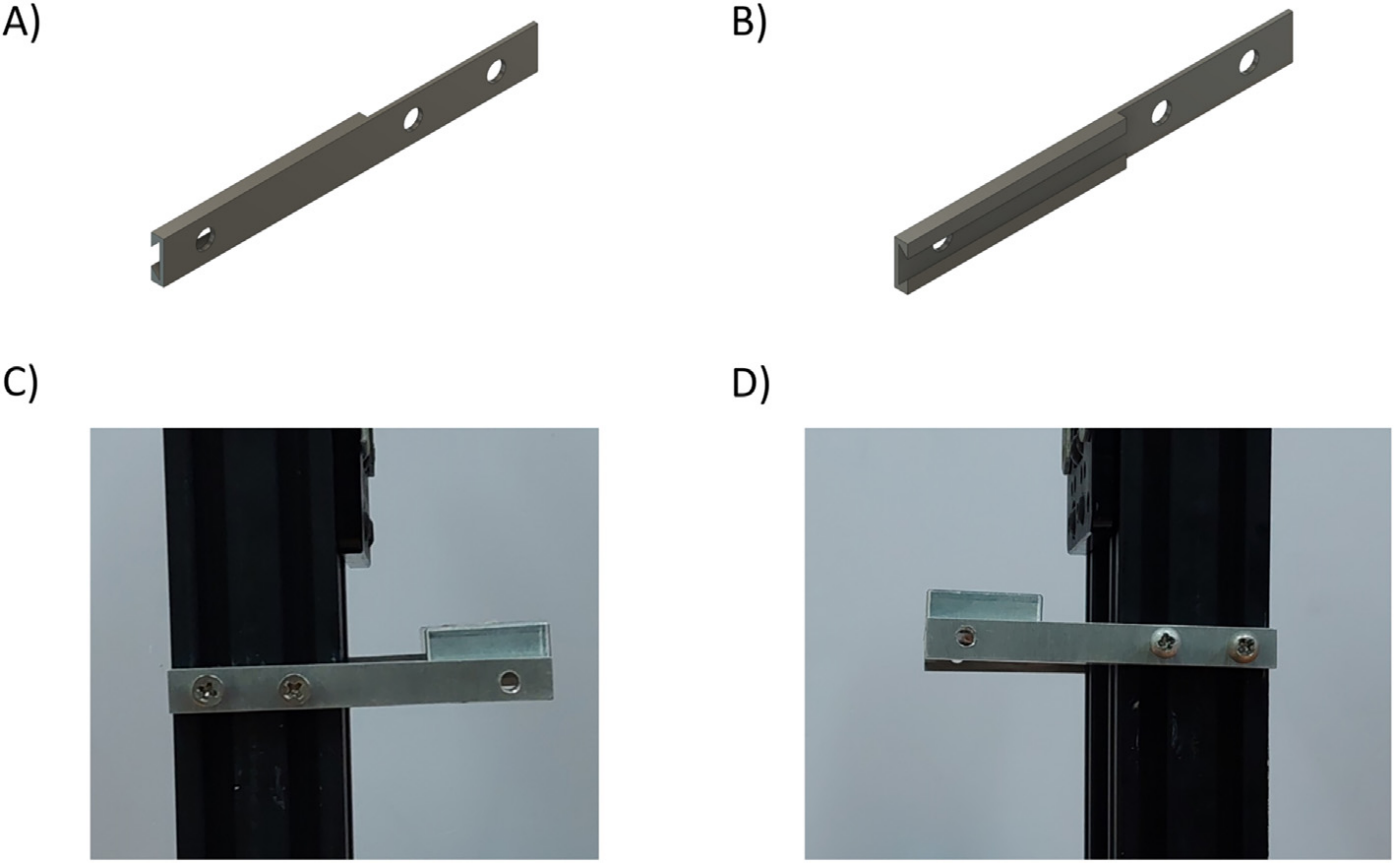
CNC cutting of (A) Right Sliding Plate Holder and (B) Left Sliding Plate Holder; Real Image and Location of (C) Right Sliding Plate Holder and (D) Left Sliding Plate Holder.

**Fig. 11:**
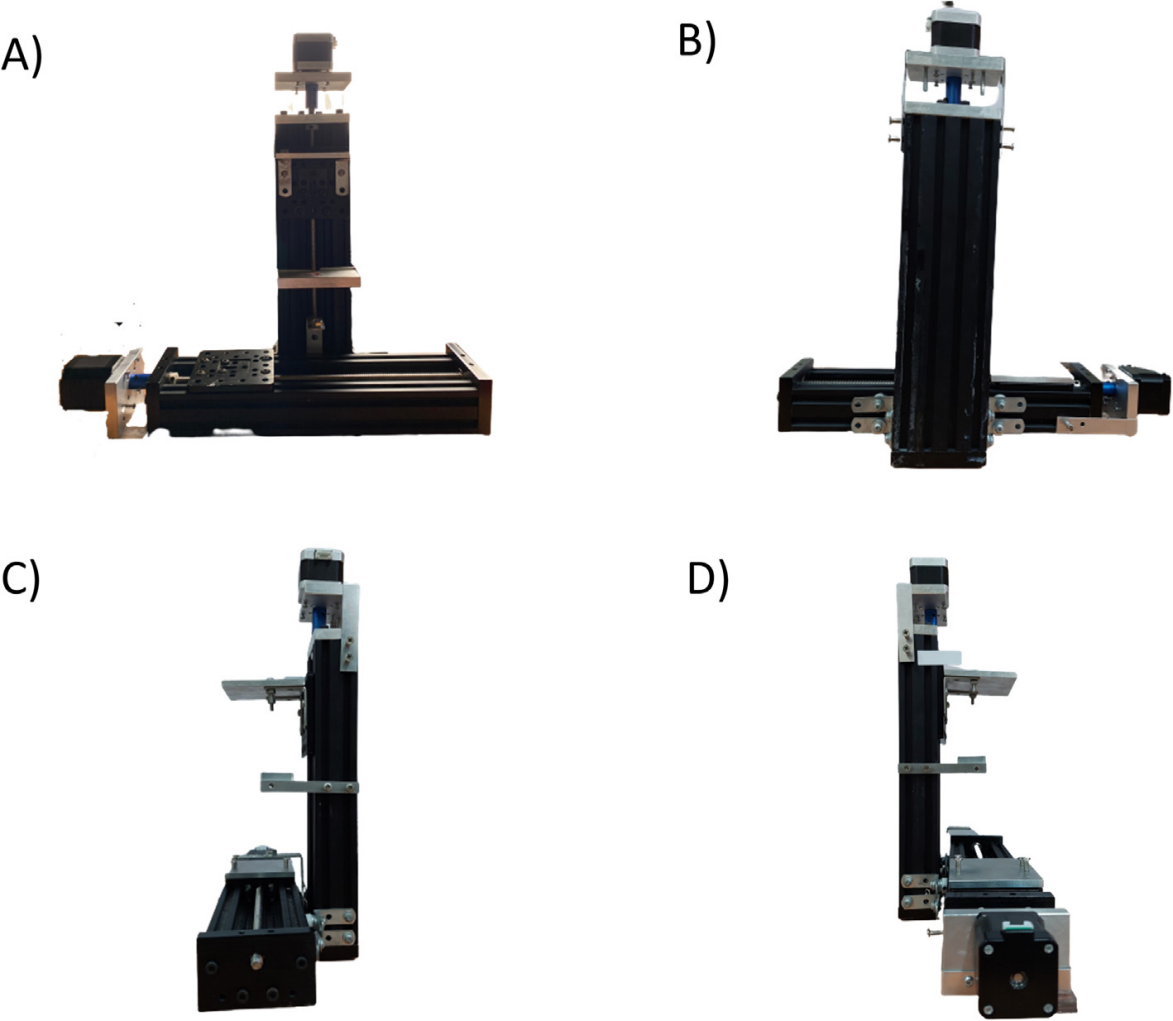
Image of SALAD in 4 different angles (A) Front-rear Angle, (B) Behind Angle, (C) Right-side Angle, (D) Left-side Angle.

Besides the two available linear actuators, some of the CNC Machining exterior materials were built using Fusion 360 software and cut-in aluminum materials to assist the functionality of SALAD. The cut CNC components are the *Needle Handle* (Fig. 5), which is used to stabilize the needle syringe; in addition, the Rubber-O-Ring is placed around the center hole to stabilize the syringe; the *Stepper Shelf Top* (Fig. 6), which is used for linking the Stepper Motors with the Ox and Oy Actuators, more specifically, the Stepper Motors are connected to the Stepper Shelf Top using four M3 screws while the Stepper Shelf Tops are linked to the Ox and Oy Actuators using two galvanized irons with four M4 screws for each actuator; the *Plate Pusher* (Fig. 7), which is used for pushing down the Needle Syringe and is connected to the Oy Actuators’ C-beam Gantry Plate using the two galvanized irons and two M4 screws; the *Membrane Plate* (Fig. 8), which is used for immobilizing the membrane and is connected to the Ox Actuators’ C-beam Gantry Plate using four M4 screws; the *Two-side vertical frames* (Fig. 9A-B), which are used for linking the Stepper Shelf Top with the Oy (Fig. 9.C) and Ox actuators (Fig. 9D); the *Two-side sliding plate holders*, which are used for connecting the Needle handle with the Oy actuators (Fig. 10). In addition, a galvanized iron is attached to the middle of the Ox and Oy Actuators, which serves the purpose of holding the needle part of the syringe pump (Fig. 11). After completion with the setup (Fig. 11), the length for the SALAD, starting from the Stepper Top Shelf to the end of the Actuators will be 340 mm for both Ox and Oy axis.

## Operation instructions

### Operation Scenario

*Note: The total rotating time is defined as the rotating duration of the first stepper motor. The delay time is the lagging between the second stepper motor initiation and the first. In addition, the speed at which Stepper 1 and Stepper 2 rotate can be modified even when they are rotating for the purpose of examining the most optimal rotating speed. In addition, the rotating speed value displayed on the LCD Screen is on a 1*–*10 scale (with 1 being the lowest speed and 10 being the highest speed).*

**Fig. 12:**
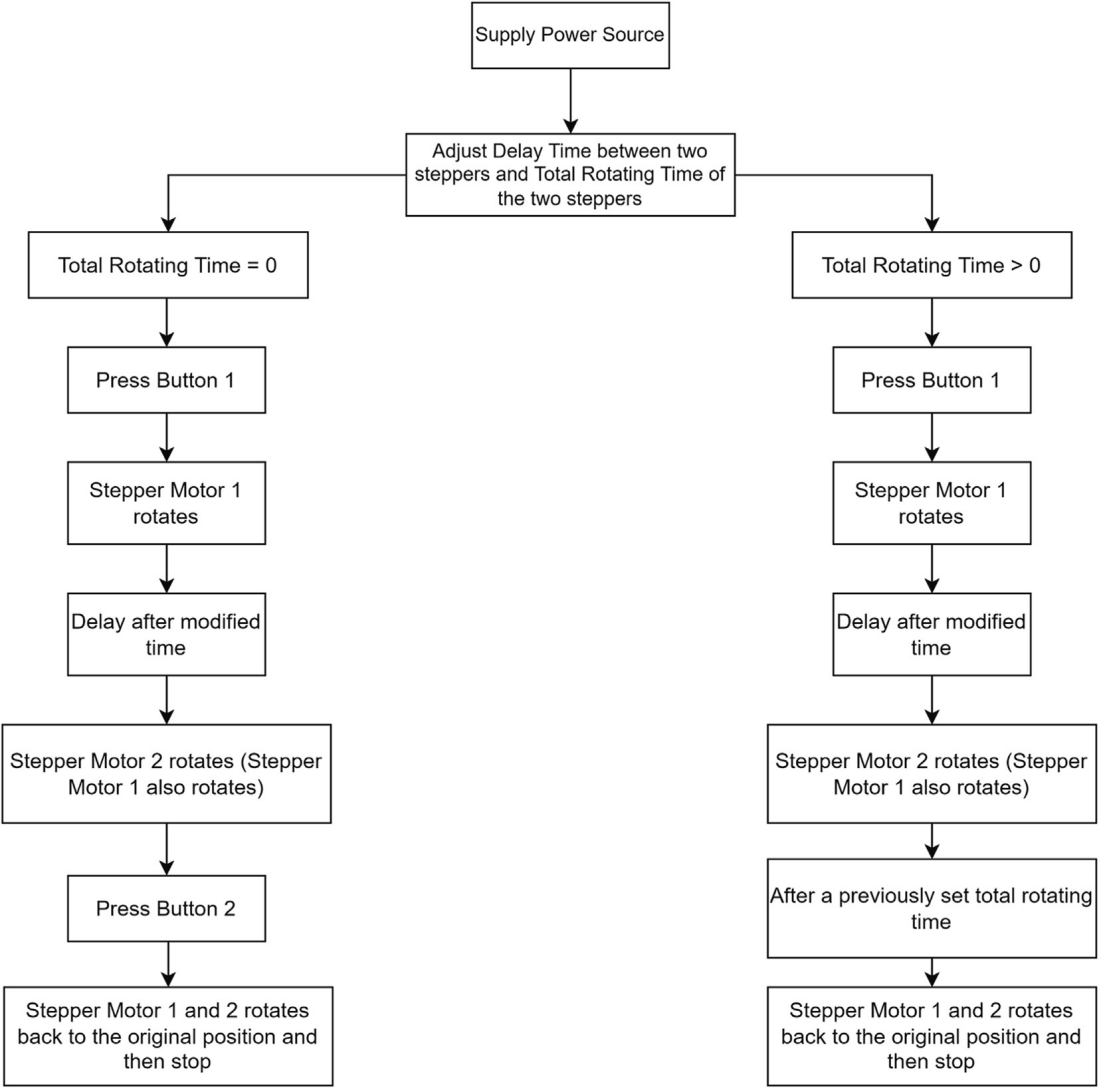
The Operation of Two Buttons.

**Scenario 1 - When the total rotating time is set equal to 0 (Rotating Time = 0) (**Fig. 12).

When Button 1 is pressed, stepper 1 rotates to make the plate move in a vertical direction (y-axis). After a modified delay time, stepper 2 spins concurrently with stepper 1, making the plate move in a horizontal direction (x-axis). After observing the line printed on the test strip, the stepper motors would be rotated back to their original position *manually by pressing button 2*.

## Scenario 2- when the total rotating time is set>0 (Rotating Time > 0) (Fig. 12)

When Button 1 is pressed, Stepper 1 rotates to make the plate move in a vertical direction (y-axis). After a modified delay time, Stepper 2 spins simultaneously with Stepper 1, making the plate move in a horizontal direction (x-axis). Next, the two stepper motors rotate back to their original position *after a previously set rotating time*. It should be taken into account that in this scenario, if we want to stop the machine immediately, Button 2 ought to be pressed.

In addition, the speed of two stepper motors can be adjusted using the two potentiometers before and during the rotation of the two stepper motors. To demonstrate how the line dispenser works more clearly, a video demonstration of Scenario 2 can be found in the online repository.

### Clarification for separating the two scenarios

*Scenario 1:* Manual Control is required. Stop time is needed to be preset for the two stepper motors. It will help us to test how long the full-total rotating time can be modified for different metrics of the test strips (For e.g., different lengths and widths of the printed line). Two buttons are needed to push in this case for the whole Line Dispenser operation to work fully.

*Scenario 2:* It only requires us to set the full-total rotating time before the system operates; therefore, it will save time when one already knows how much time is needed for the line to be fully printed on the test strip. Only one button is needed to press in this case for the whole Line Dispenser operation to work fully.

### Operation manual

**Fig. 13:**
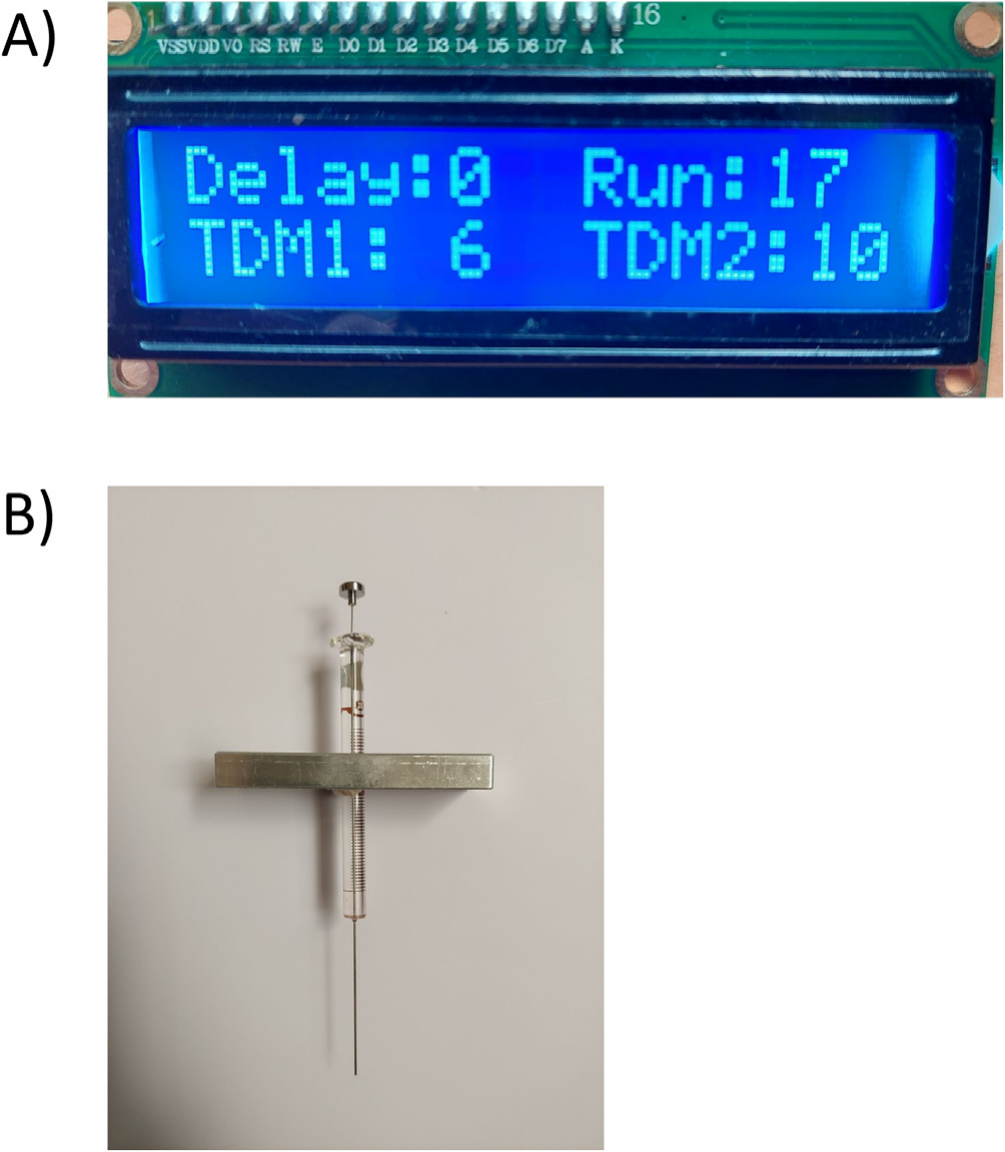
(A) LCD Screen with Four Modifiable variables (Delay time, Running time, Speed of Stepper Motor 1, and Stepper Motor 2). (B) Placing the Needle Syringe into the Needle Handle.
Place the test strip onto the metal plate (Ox Actuator)
Pump the reagent into the Syringe Pump and attach the Syringe Pump to the Needle Handle (Fig. 13B)
Place the Needle Handle onto the Sliding Plate Holder (Fig. 14)

Check the wiring of the two stepper motors to the control board (4 pins JST-PH cables are connected to the four pins of the Stepper Motors)
Connect the DC jack to the 12 V-5A Power Supply and Connect the Power Supply to the power source
Turn SALAD on by pressing the “Powering” button
Adjust the speed of the y-axial stepper motor (TDM1; value range from 1 to 10); the speed of the x-axial stepper motor (TDM2; value range from 1 to 10); the delay time between Motor 1 and M2 (Delay; secs; = t1- t2) and the total run time (Run; secs; =t1), which can be seen in the LCD Screen. (Fig. 13A)

**Fig. 14:**
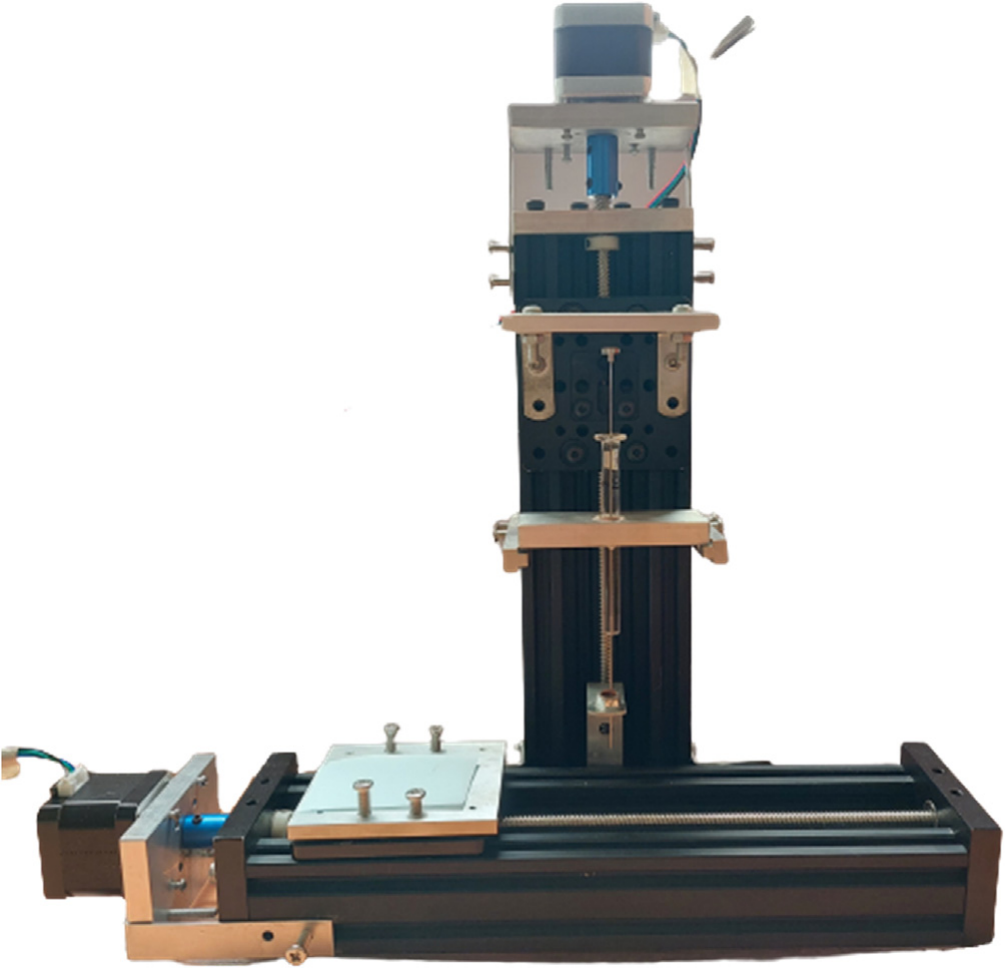
Full Setup of SALAD.
Press Button 1 or 2 and rotate the potentiometers, which depend on the type of operation that needs to be executed.

## Validation and characterization

### Evaluation experimental design

**Fig. 15:**
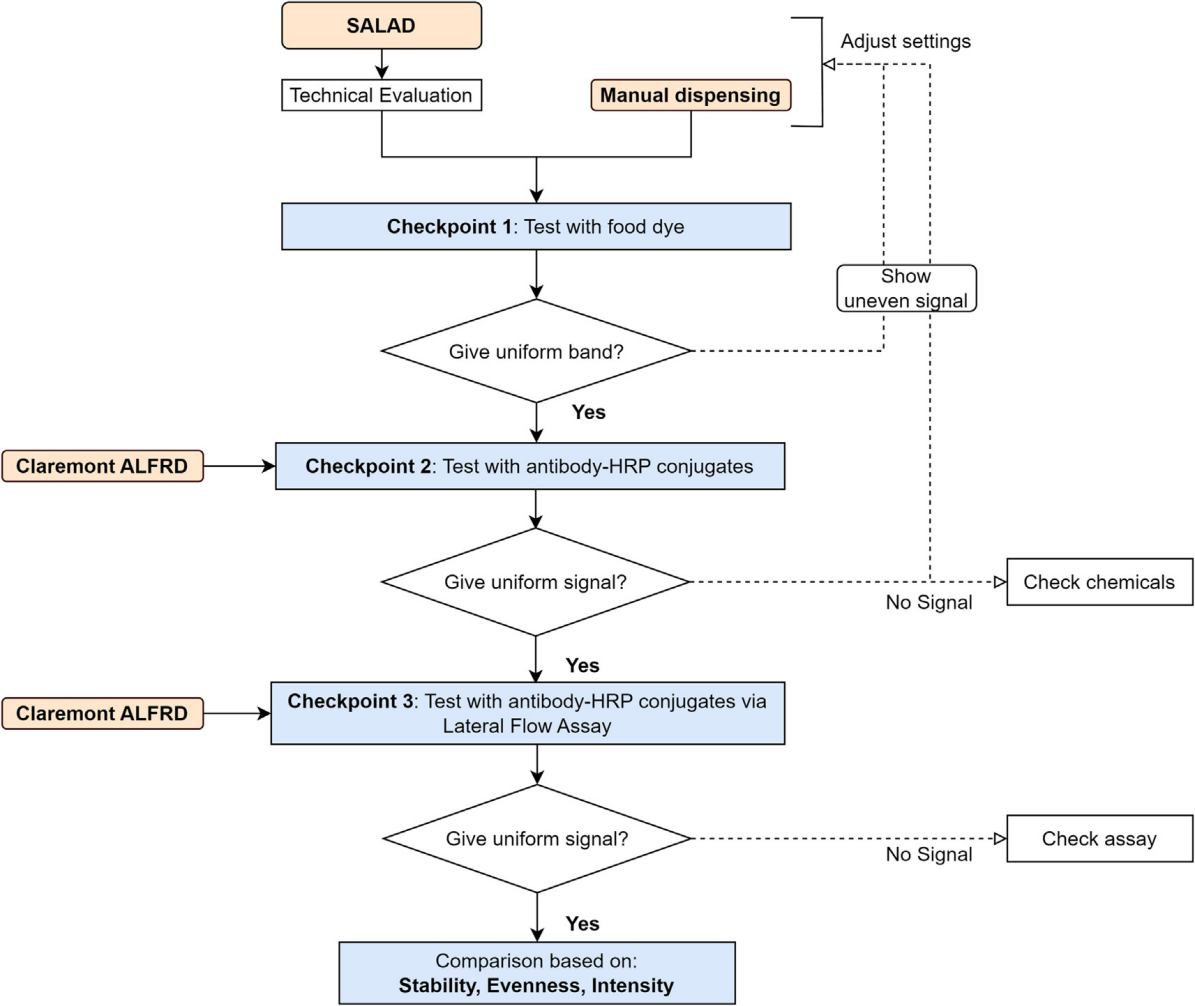
Study design of the evaluation process.

After the initial technical evaluation to collect information regarding speed, output volume stability, and input range (volume, voltage, ampere), there were three checkpoints to evaluate the performance of our proposed method thoroughly. The first checkpoint is to visually assess the evenness of the band formed with food dye. The second checkpoint is to check uniformity in dispensed IgG-antibody [HRP] conjugates, followed up by the tertiary checkpoint to evaluate the uniformity of signal under the effects of lateral flow assay (Fig. 15). At each point, if the result is not optimal, chemical validation and system calibration would proceed.

### Evaluation materials

**Fig. 16:**
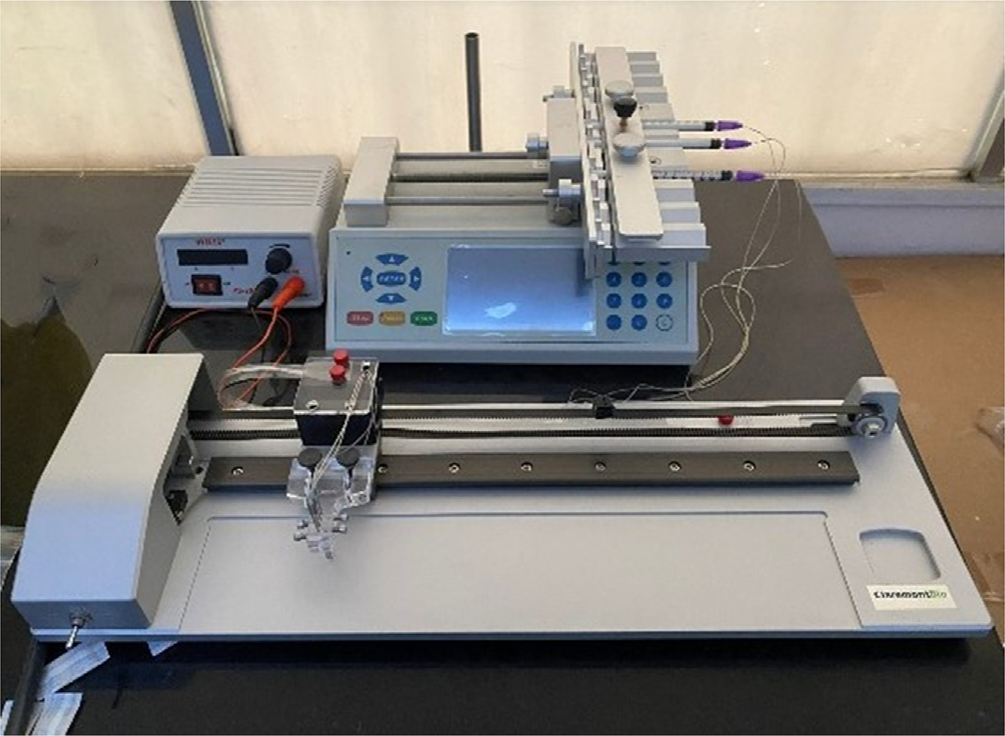
Claremont Bio Automated Lateral Flow Reagent Dispenser (Claremont ALFRD).

Nitrocellulose Membrane, 0.45 μm (#88018, Invitrogen), Whatman Standard 14 as the sample pad, and Whatman CF4 as the absorption pad were used to build the assay platform. The food dye solution (local market) was utilized in checkpoint #1 evaluation at 1:5 dilution (Fig. 15). Cortisol Polyclonal antibody (#PA1-85347, Invitrogen) and Anti-Sheep [HRP] antibody (#61-8620, Invitrogen) was used in our evaluation assay at checkpoint #2 and #3 (Fig. 15). The antibodies were diluted in phosphate buffer PBS 1X. BSA 5 % was used as a blocking reagent. PBS 1X was used as a reagent-dilution buffer, and PBS-T (0.05 % Tween: PBS 1X) as a washing buffer. Additional equipment required includes an orbital shaker, vortex, and Azure c400 Visible-Fluorescence Western Blot Imaging System. The evaluation standard in this study was Claremont Bio Automated Lateral Flow Reagent Dispenser (Claremont ALFRD, https://www.claremontbio.com/Lateral-Flow-Reagent-Dispensers-s/114.htm), which implements a similar working principle as ours (Fig. 16).

### Evaluation protocol

#### Membrane assembly

Lateral flow wick is assembled by adhering the sample pad and absorption pad on top of the nitrocellulose membrane at two ends, respectively, following the design described in [19].

#### Manual immobilization method

The implemented manual method was designed based on the working principle of a dispenser and the discussed foundation of protein-nitrocellulose interaction: A microneedle was used to dispense reagents. This method mainly relied on the small head size of the injectors (approx. 0.01 – 0.1 mm), to form micro-droplets and the passive fixation ability of IgG antibodies on the nitrocellulose membrane on contact without additional catalysts [12], [20].

#### Testing with dye

The food dye solution was dispensed onto nitrocellulose membranes using two dispensing methods: Manual methods and SALAD. This step is to evaluate the dispensing efficiency visually. 3 μL of food dye was dispensed per sample.

#### Immunoassay testing

Anti-Sheep [HRP] antibodies (0.2 μL/mL) was immobilized onto nitrocellulose membrane using three dispensing methods, including manual approach, SALAD and Claremont ALFRD to evaluate the dispensing efficiency for biological assays in checkpoint #2. Three μL of antibodies were dispensed per sample. The dispensing rate was set at 0.3 μL/s for Claremont ALFRD and M1 = 5 for SALAD. After one hour of incubation at room temperature (RT), they were soaked in blocking solution for one hour at RT and washed twice again, 5 mins each at 80 rpm, before being added with luminol.

To evaluate the stability of immobilized antibodies under lateral flow effects, the Cortisol Polyclonal antibody (50 μL/mL) was immobilized initially in the nitrocellulose region as the primary antibody using two dispensing models: Claremont and SALAD. Three μL of Cortisol Polyclonal antibody were dispensed per sample. The dispensing rate of Claremont ALFRD and SALAD, the blocking step, and the washing step were set similarly to what was in the experiment with Anti-Sheep [HRP] antibodies. The membrane was then left to dry at RT for one hour and assembled with a sample pad and conjugate pad. Sequentially, 200 μL of Anti-Sheep [HRP] antibody solution (1:4000 dilution) would be added onto the sample pad, letting it flow for 20 mins. The wick would then be washed twice for 5 min each at 80 rpm. Finally, the wick would be added with luminol.

The added luminol generates chemiluminescence by reacting with HRP and can be detected with Azure c400 Visible-Fluorescence Western Blot Imaging System. The imaging settings were normal sensitivity, exposed for 1 min.

#### Image analysis

Acquired imaging results would be imported to ImageJ - NIH [21] for further analysis, including (1) area measurement (width × length), (2) Mean signal intensity, (3) pixel densitometry – pixel distribution over the scale of intensity. The signal band was manually identified for the measurement. After three measurements were collected, statistical analysis proceeded to compare results yielded by different dispensing methods. Independent t-tests and descriptive tests were conducted between Claremont’s and SALAD’s results within each measurement. Regarding pixel densitometry, kurtosis, and skewness were used to analyze the curve distribution. For the signal to be even, there would be more pixels accumulated at one certain peak instead of scattering over the scale. Henceforth, kurtosis and skewness are expected to be higher if the signal is even [22]. Signal-to-Noise ratio (SNR) is also calculated following the equation [23]:

$$\mathrm{SNR}=\frac{\mathrm{Average}\;signal}{\mathrm{Minimum}\;signal\;(Background\;signal)}$$

Sequentially, an independent *t*-test would be done for SNR.

### Evaluation results

We conducted a comprehensive evaluation, as described in Fig. 15, to confirm the stability of the prototype in comparison to the manual dispensing using micropipettes and the commercialized dispenser Claremont ALFRD via three checkpoints. Overall, the prototype reached a production success rate of 80% (eight out of ten membranes printed successfully), while the Claremont ALFRD has a success rate of approximately 90% (approximately nine out of ten membranes printed successfully).

#### Checkpoint 1: Test with blood dyes

**Fig. 17:**
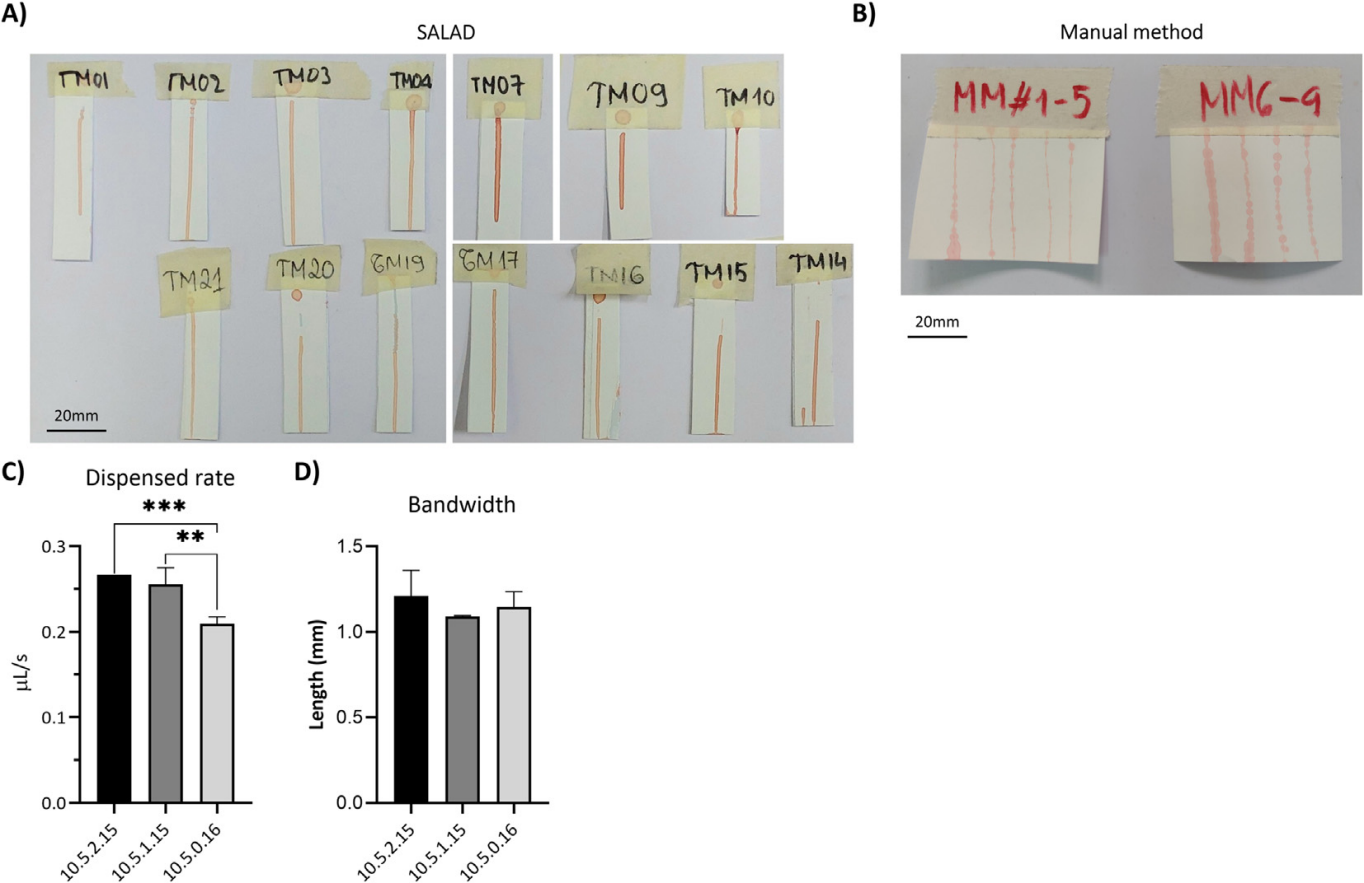
Checkpoint 1 results from SALAD (n = 14), manual methods (n = 9). Imaging results from (A) SALAD, (B) Manual methods: Comparison amongst three SALAD settings – 10.5.2.15 (n = 4), 10.5.1.15 (n = 3), 10.5.0.16 (n = 4) in (C) dispensed rate and (D) bandwidth. The name of the settings was labeled following this principle: M2 - M1 - delay - total runtime).

In the first checkpoint, SALAD was compared with a traditional manual approach to see if it could improve the effectiveness of reagent dispensing, using food dye as a preliminary test reagent. Dispensing results of SALAD exceeded the results yielded by the manual approach, notably in evenness and bandwidth uniformity (Fig. 17A, B). The manual results showed clear inconsistency in bandwidth, which was likely due to the lack of control over the dispensed rate of this method. Within the exact setting, there was no significant variance detected (Fig. 17C). At the same dispensed rate setting (M1 = 5; M2 = 10), there were significant differences in the actual dispensed rate caused by different delay and run time (delay = 0, run time = 16, 10.5.0.16) with the other two settings (delay = 1, run time = 15, 10.5.1.15; delay = 2, run time = 15, 10.5.2.15) (p = 0.001 and p < 0.001, respectively) (Fig. 17C). The bandwidth yielded by the similar dispensing rate settings remained comparable (Fig. 17D). Hence, it can be deduced that the dispensed rate of SALAD is moderately stable, yet adjustments in other parameters like run time and delay could slightly affect the dispensed rate. To further evaluate whether this level of instability is acceptable for research purposes, SALAD was next compared with the commercialized model - Claremont ALFRD.

#### Checkpoint 2: Test with antibody-HRP conjugates

**Fig. 18:**
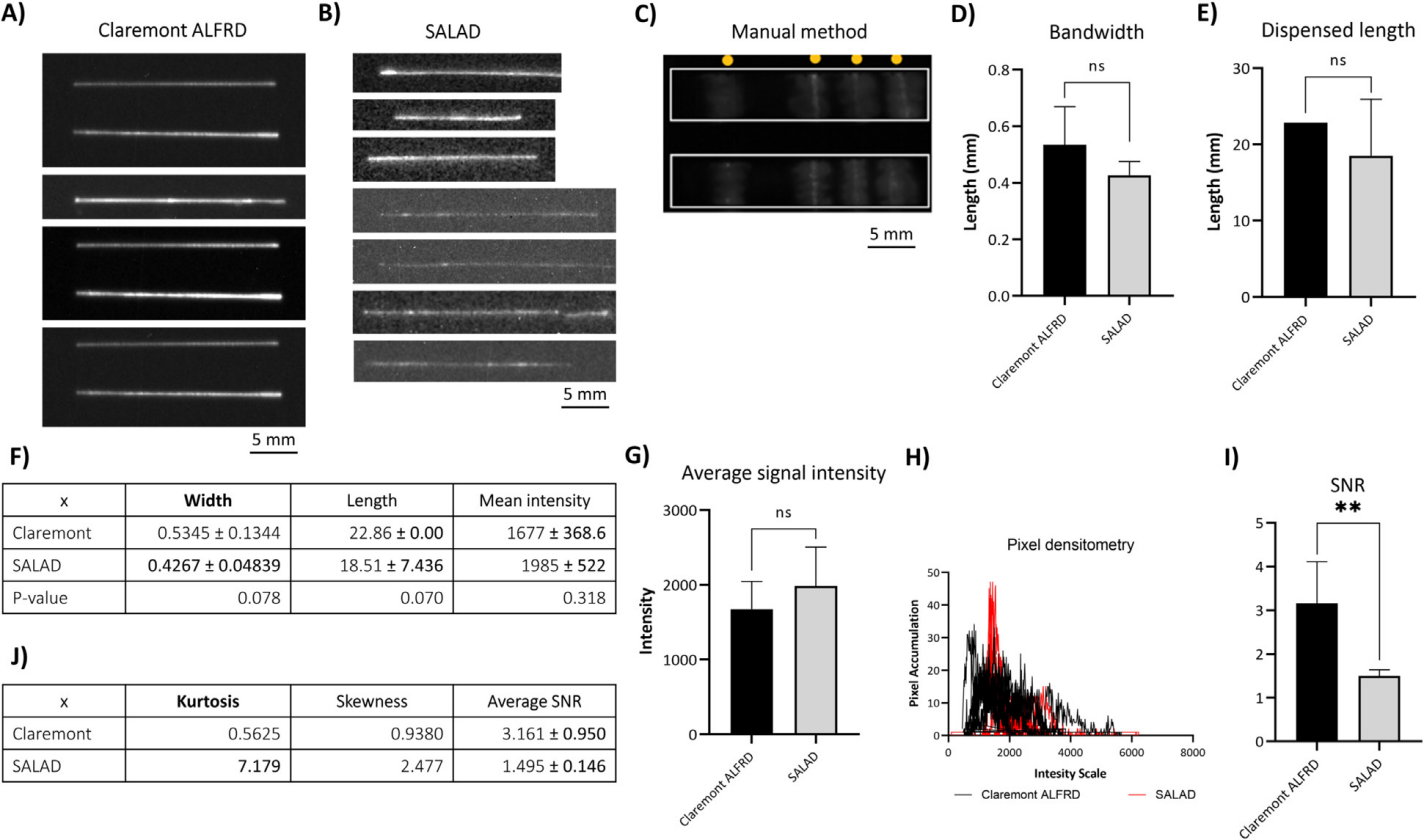
Checkpoint 2 results from Claremont ALFRD (n = 7), SALAD (n = 7), manual methods (n = 8): Imaging results from (A) Claremont, (B) SALAD, (C) Manual methods (yellow dots indicate the position of antibody band); Comparison between Claremont and SALAD in (D) bandwidth and (E) dispensed length; (F) Table showing descriptive statistics (Mean ± Std) of Claremont and SALAD in bandwidth, length, and average signal intensity; Comparison between Claremont and SALAD in (G) average signal intensity, (H) pixel densitometry, (I) SNR; (J) Table showing descriptive statistics (Mean ± Std) in pixel densitometry’s kurtosis and skewness, and their SNR. (For interpretation of the references to color in this figure legend, the reader is referred to the web version of this article.)

Checkpoint #2 then proceeded to investigate further the evenness of biosignals produced by antibodies dispensed by different approaches. These results will reinstate the prototype’s functionality on biological reagents. Both models yielded fair results with undetectable smearing or unevenness, outperforming the manual approaches - which has visible smearing with notably uneven bands (Fig. 18A-C). The manual approach was eliminated from the evaluation at this point due to its poor results. At the same dispensing rate (approx. 0.3 μL/s) and conveyor speed (approx. 11 mm/s), both models showed no significant differences in either bandwidth (Fig. 18D & Table 18F, p > 0.05) or total dispensed length (Fig. 18E & Table 18F, p > 0.05). However, within each parameter, differences in their standard deviations (Std) are worth noticing (Table 18F). For the bandwidth parameter, Claremont’s Std is approximately three-fold SALAD’s Standard Deviation (0.1344 and 0.0484, Fig. 18D, respectively), indicating less stability in the syringe pump compartment on Claremont’s behalf. On the contrary, Claremont showed no variance in the length parameter, while SALAD has a comparatively high Standard Deviation (7.436), which implies instability in SALAD’s conveyor belt motions (Fig. 18E). The mean signal intensity, representing the amount of antibodies successfully immobilized onto the nitrocellulose membrane, displayed no differences between the two models (Fig. 18G, p > 0.05). Regarding the evenness, SALAD tends to exceed the performance of Claremont slightly. The accumulation of pixels within imaging results is found at adjacent intensity values to the peak of the histogram for both models (Fig. 18H). This trend is more cogent in SALAD’s results. According to Table 18J, SALAD yielded comparatively higher measurements in skewness and kurtosis (two-folds and 14-folds, respectively), implying better evenness in signals created by dispensed antibodies. On the other hand, SNR in SALAD’s is lower than that of Claremont (Fig. 18I, p = 0.001), suggesting SALAD dispensed fewer antibodies onto the membrane than Claremont did. Yet the injection stability of SALAD is once again confirmed by the lower Std and SNR compared to that of Claremont (Table 18J, 0.146 and 0.950, respectively). In general, SALAD showed little disadvantage in evenness or stability compared to Claremont ALFRD, yet modifications in the conveyor belt can be considered to achieve better-dispensed length stability.

#### Checkpoint 3: Test with antibody-HRP conjugates via lateral flow assay

**Fig. 19:**
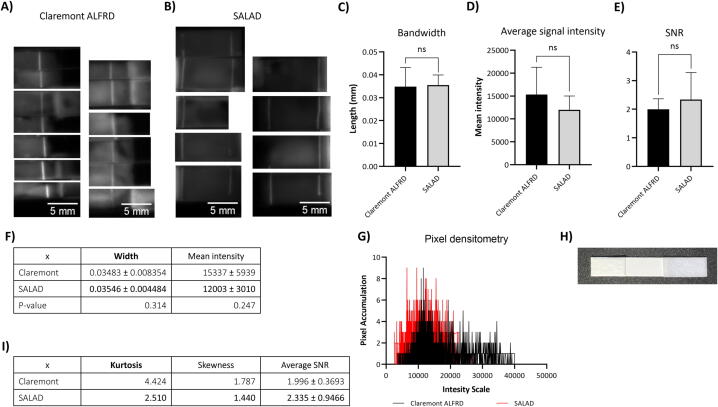
Checkpoint 3 results from Claremont ALFRD (n = 12) and SALAD (n = 13): Imaging results from (A) Claremont, (B) SALAD; Comparison between Claremont and SALAD in (C) bandwidth, (D) average signal intensity and (E) SNR; (F) Table showing descriptive statistics (Mean ± Std) of Claremont and SALAD in bandwidth and mean signal intensity; Comparison between Claremont and SALAD in (G) pixel densitometry; (H) Assembly of the lateral flow wick; (I) Table showing descriptive statistics (Mean ± Std) of Claremont and SALAD in pixel densitometry’s kurtosis and skewness, and their SNR.

Checkpoint #3 was then conducted to testify to the stability of immobilized antibodies post-affected by capillary forces of lateral flow assay, whose assembly is demonstrated in Fig. 19H. Both models yielded fair results with an even band and outstanding signal at the band compared to the background noises (Fig. 19A-B). However, higher background noises can be noticed in Claremont's results. Both models showed no differences in either bandwidth (Fig. 19C & Table 19F, p > 0.05) or mean signal intensity (Fig. 19D & Table 19F, p > 0.05). However, the differences in their bandwidth Std reaffirmed the higher injection stability given by SALAD (0.0045 and 0.0084, respectively). In contrast to the SNR results yielded in checkpoint #2, in this experiment, SALAD achieved comparable SNR with that of Claremont (Fig. 19E & Table 19H, p > 0.05). Both SALAD and Claremont showed comparable evenness as the accumulation of pixels within their imaging results is found at adjacent intensity values to the peak of the histogram (Fig. 19G). This trend is slightly more cogent in Claremont’s results, as their peak is sharper. According to Table 19I, both models achieved similar measurements. In summary, SALAD and Claremont showed no differences in evenness or bandwidth. However, the contrary in Claremont’s SNR required further discussion in the following section.

## Discussion

In this paper, we developed a low-cost syringe-based line dispenser using the Arduino UNO board (SALAD) and compared its functionality with a manual approach and commercialized products. SALAD yielded comparable performance with Claremont (Claremont ALFRD) and outperformed manual methods regarding time efficiency, labor intensity, and quality of the dispensed lines (Table 1). However, there was a notable variation in the length parameter of SALAD, indicating instability in the stepper motor-driven conveyor belt (Fig. 18E). This is caused by current ripple and step loss of the device motor leading to non-uniform angular increments and output torque [24], [25]. Subsequently, loss of motion synchronization increases vibration and causes motor run-by-run inaccuracy. This phenomenon can be restricted by reducing the micro-stepping size and lowering the excitation energy delivered to the motor coil, which results in less vibration and more stable rotor movement [26]. Another approach is to install a mechanical damper on the stepper motor shaft and add extra inertia to each step, which helps to absorb the excess electrical flux and provide a smoother micro-stepping effect [27]. Nevertheless, motor vibration and oscillation have been a significant concern in low-cost device development since the manufacturer has to consider between the stepper motor’s cost and its performance.

**Table 1:** Comparisons between three methods: Manual immobilization, Claremont ALFRD, and SALAD.

		Manual immobilization	Claremont ALFRD	SALAD
Duration	Setting up	< 5 mins	15–20 mins	5–10 mins
	Proceed	Approx. 5 mins	1–2 mins	1–2 mins
Cost		Free of charge	$4,750.00	$200.61
Evenness		+	+++	+++
Labor intensity		++++	+	++
Overall efficiency		+	++	+++

On the other hand, both dispensers showed comparable results in the line evenness and the amount of antibody immobilized (Fig. 18H & Fig. 19G). Lower background noises and a slightly better SNR were observed in SALAD’s strips when compared with Claremont ALFRD’s (Fig. 19E). Even though Claremont was originally shown to dispense more antibodies onto the membrane, there was an increase in background noises after the flow, implying the dispensed antibodies had been displaced due to capillary forces (Fig. 19A). This phenomenon might lead to a reduction in its SNR (Fig. 19E). The stability of antibodies dispensed by SALAD was hypothesized to be the result of a more optimal contact angle (90°) between the dispensed antibodies and the membrane, or the needle and the membrane [28], [29]. The direct contact angle increases friction force between the needle and the membrane and, with an appropriate dispensing rate, enhances the antibodies line evenness.

Apart from commercial products, the number of articles aimed at dispensing reagents in an automated manner with low-resource settings is limited. Won Han and Joong Ho Shin developed a low-cost and 3D-printed antibody dispenser using the Arduino microcontroller and dual DC motors [12]. The needle of their device is bent at an angle similar to Claremont ALFRD's and still achieves a comparable evenness with SALAD. However, their design used the DC motors and L298N motor driver to pass the nitrocellulose membrane through the fixed needles [12]. Despite their low cost, L298N and DC motors are unstable and generate considerable heat during operation, thus decreasing the device's overall lifetime. This requires frequent dispenser maintenance and is inappropriate for medium-to-large LFA strip experiments. Therefore, we utilized the TB6600 Driver and stepper motor in our dispenser for more durability and stability. Moreover, the paper validated the lines' signal intensity using only the background contrast color and did not fully characterize its variability [12]. In this paper, we proposed a pipeline to effectively analyze the signal intensity of dispensed reagent lines combining line bandwidth, signal-to-noise ratio, average signal intensity, and signal intensity pixel densitometry. With this, we expected to establish a guide for analyzing the signal intensity variation in future automated line dispenser research, especially for low-cost and lab-scale antibody dispensers.

While several limitations still need to be addressed, such as the variance in the conveyor belt (Ox Actuators) speed, SALAD can minimize the complication of producing POC/LFA in limited-resource settings. Firstly, unlike commercialized products that are expensive, SALAD can be easily manufactured with low-cost and easy-to-find materials. Both the Arduino UNO, the TB6600 driver, and the stepper motors are available on any online marketplace and can be easily purchased. With a simple design, this model can also be maintained periodically without professional help. Secondly, utilizing an automated line dispenser can reduce labor intensity and assist in the mass production of POC devices Finally, SALAD offers the benefit of providing a moderate system footprint for small to medium-sized laboratories, highlighting the applicability of SALAD in developed and developing countries (SALAD: 380 × 380 × 128 mm; Claremont: 457 × 146 × 75 mm without source and syringe pump; Syringe pump #Z401366: 228 × 152 × 127 mm). Therefore, the model introduced by this study represents significant progress toward the investigation of POC diagnostic devices. As a stand-alone device with offline performance and a strictly followed loaded program, SALAD is immune from online data breaches - one of the top concerns in biomedical security [30]. However, interference and overwriting still pose a threat to the security of stand-alone devices like SALAD. Henceforth, the following section will discuss the latent developments to optimize the prototype.

The dispensers will be further optimized in future studies to enhance their performance and minimize motor instability. Alternative microcontrollers with higher resolution, such as Microchip AVR 16–32 bit or XIAO (SAMD21) 32-bit, can replace the 8-bit microcontroller used in this study for better stability. With an updated controller, lock bits can be included to protect the code flow from overwriting and interference. To surmount the instability occurring in the conveyor belt, more powerful stepper motors with higher torque, such as NEMA 23 and 27, can replace the original NEMA 17 to overcome the resistance in the belt. Position switches can be added to both axes of the prototype to adjust the needle handle’s position automatically, avoiding overrun and calibrating the system automatically. Furthermore, more advanced stepper motor drivers, such as the DM542 Driver Leadshine, can be utilized to ensure smoother and less noisy stepper motors while running, resulting in higher efficiency. In addition, the diameter of the syringe needle determines the flow resistance through its opening and affects the line width [12]. Theoretically, smaller syringe needles will provide a nominal contact area between the syringe and minimize smearing. Thus, SALAD will be tested with different needle diameters at the same setting and investigated for the correlation between the syringe diameter and the dispensed lines’ geometry. Furthermore, POC diagnostic test strips frequently contain two dispensed lines: (1) the detection and (2) control lines with different reagents for each line. With further improvements, SALAD can be upgraded to print multiple reagent lines simultaneously to shorten manufacturing time.

## Conclusion

An Antibody Dispenser using the Arduino system and a Syringe (SALAD) has been demonstrated in this paper, with which the final purpose is to build a line dispenser that is low-cost and can create an appropriate line on the membrane surface. The system consists of Arduino UNO, two stepper motors, two motors drivers, two stepper motors actuators, and an LCD screen with supplement instruments (buttons, power supply, syringe pump), which serves the goal of building an economic system and can be operated through the two scenarios, depending on the users’ purposes. Using the three evaluation methods (testing with food dyes, antibody-HRP conjugates, and antibody-HRP conjugates through Lateral Flow Assay) and making the comparison with a commercialized model and a manual method, the SALAD has shown to be more effective than the manual method and has similar outcomes when comparing with the Claremont ALFRD commercialized model through different results (Kurtosis, Skewness, Signal Intensity, Bandwidth, Dispense length, Signal-to-Noise ratio, Width, Pixel densitometry). Finally, further work to enhance the stability of the models is required, such as changing the microcontrollers and changing the stepper motor “drivers. With the current production cost and feasible design, this model is expected to ease the burdens for institutions that are interested in fabricating lateral flow assays with a limited budget.

## Declaration of Competing Interest

The authors declare that they have no known competing financial interests or personal relationships that could have appeared to influence the work reported in this paper.
